# Regulation of MRP4 Expression by circHIPK3 via Sponging miR-124-3p/miR-4524-5p in Hepatocellular Carcinoma

**DOI:** 10.3390/biomedicines9050497

**Published:** 2021-04-30

**Authors:** Haihong Hu, Yu Wang, Zhiyuan Qin, Wen Sun, Yanhong Chen, Jiaqi Wang, Yingying Wang, Jing Nie, Lu Chen, Sheng Cai, Lushan Yu, Su Zeng

**Affiliations:** Cancer Center of Zhejiang University, Zhejiang Province Key Laboratory of Anti-Cancer Drug Research, Institute of Drug Metabolism and Pharmaceutical Analysis, College of Pharmaceutical Sciences, Zhejiang University, Hangzhou 310058, China; huhaihong@zju.edu.cn (H.H.); 21919028@zju.edu.cn (Y.W.); qinzy20@zju.edu.cn (Z.Q.); sunw127@zju.edu.cn (W.S.); hihollychen@zju.edu.cn (Y.C.); wangjqzs@zju.edu.cn (J.W.); 3140100174@zju.edu.cn (Y.W.); niejing@zju.edu.cn (J.N.); 11419030@zju.edu.cn (L.C.); caisheng@zju.edu.cn (S.C.)

**Keywords:** hepatocellular carcinoma, multidrug resistance, circHIPK3, miR-124-3p, miR-4524-5p, MRP4

## Abstract

Multidrug resistance-associated protein 4 (MRP4), a member of the adenosine triphosphate (ATP) binding cassette transporter family, pumps various molecules out of the cell and is involved in cell communication and drug distribution. Several studies have reported the role of miRNAs in downregulating the expression of MRP4. However, regulation of MRP4 by circular RNA (circRNA) is yet to be elucidated. In this study, MRP4 was significantly upregulated in hepatocellular carcinoma (HCC) tissues compared to the adjacent noncancerous tissues. Computational prediction, luciferase reporter assay and miRNA transfection were used to investigate the interaction between miRNAs and MRP4. miR-124-3p and miR-4524-5p reduced the expression of MRP4 at the protein but not mRNA level. Circular RNA in vivo precipitation and luciferase reporter assays demonstrated that circHIPK3, as a competitive endogenous RNA, binds with miR-124-3p and miR-4524-5p. Further, knockdown of circHIPK3 resulted in downregulation of MRP4 protein, whereas cotransfection of circHIPK3-siRNA and miR-124-3p or miR-4524-5p inhibitors restored its expression. In conclusion, we report that miR-4524-5p downregulates the expression of MRP4 and circHIPK3 regulates MRP4 expression by sponging miR-124-3p and miR-4524-5p for the first time. Our results may provide novel insights into the prevention of MRP4-related proliferation and multiple drug resistance in HCC.

## 1. Introduction

Hepatocellular carcinoma (HCC) is estimated to be the sixth leading cause of cancer incidences and fourth leading cause of cancer-related deaths worldwide [1]. Radical resection and transplantation of liver are the first line of preferred treatments for HCC. However, not every HCC patient is suitable for surgery. Hence, chemotherapy is one of the primary treatment options for HCC. However, multiple drug resistance (MDR) in HCC is believed to be a major clinical obstacle to effective chemotherapy [2]. The drug resistant tumors usually metastasize to distant organs and show poor prognosis [3]. Drug resistance can be classified into two categories: inherent resistance and acquired resistance; HCCs with the latter type are the most difficult to treat. Furthermore, MDR in the majority of the cases can be attributed to the dysregulation of genes coding for drug transporters. For instance, overexpression of the efflux transporters leads to reduction in uptake of anticancer drugs, such as adriamycin [4] and 5-fluorouracil (5-FU) [5].

Adenosine triphosphate (ATP) binding cassette (ABC) transporters are classic efflux transporters that are grouped into seven subfamilies, from ABC-A to -G. Except for the well-known ABC subfamily B member 1 (ABCB1/P-glycoprotein(P-gp)) and ABC subfamily G member 2 (ABCG2, also known as breast cancer resistance protein (BCRP)), nine of thirteen members of ABC subfamily C (ABCC, also known as multidrug resistance-associated proteins) play an important role in drug resistance [6]. Multidrug resistance-associated protein 4 (MRP4) was first discovered in 1999 in a human T-lymphoid cell line [7]. Since then, multiple studies have recognized its role in effluxing out a broad range of endogenous molecules, such as eicosanoids, conjugated steroids, urate, cyclic nucleotides and nucleotide analogues. These molecules are known to be involved in cellular communication and signaling processes, indicating that MRP4 may be a potential therapeutic target for the pathophysiological processes. Some anticancer drugs function as substrates for MRP4, which may be related to chemotherapeutic susceptibility in multiple cancers [8,9,10,11,12]. It has been shown that knocking down MRP4 using small interfering RNA reverses the acquired cisplatin resistance in gastric cancer cells [8]. Furthermore, Murray et al. reported that the suppression of MRP4 decreases the progression of neuroblastoma xenografts and increases its sensitivity to irinotecan in vivo [9].

Accumulating pieces of evidence have shown that noncoding RNAs (ncRNAs) are important regulators of cellular processes, such as cell differentiation, proliferation and metastasis, and drug resistance [13,14,15,16]. MicroRNAs (miRNAs) are 19–25 nucleotide-long regulatory ncRNAs that regulate target mRNAs and are involved in various biological functions. miR-489-3p and miR-630 suppress OCT2 expression by directly binding to its 3′-untranslated region (UTR) and decrease oxaliplatin uptake [17]. Hu et al., show in their work that the inhibition of MRP4 expression and miR-124-3p overexpression led to enhanced sensitivity to adriamicyn in MCF-7-ADR cells [18]. Long noncoding RNAs (LncRNAs) are endogenous ncRNAs that are more than 200 nucleotides long and are known to be strongly associated with the development and progression of various diseases. By altering the binding of SFPQ/E2F1/HDAC1 to the promoter region, the lncRNA SANT1 has been reported to transcriptionally regulate the expression of SLC47A2 in renal cell carcinoma [19]. With covalently closed loop structures neither having 5′3′ polarity nor a polyadenylated tail, circular RNAs (circRNAs) are more stable than linear transcripts, as they are not easily degraded by RNA exonucleases [20]. Further, multiple studies have reported the regulation of ABC transporters by circRNAs [21,22]. CircRNAs are known to be involved in gene expression and chemoresistance of tumor cells through several regulatory mechanisms, such as sponging miRNAs and modulating cell signaling pathways. Overexpression of circPVT1 has been reported to increase the expression of P-gp and contribute to doxorubicin and cisplatin resistance in osteosarcoma cells [22].

MRP4 was significantly upregulated in hepatocellular carcinoma (HCC) tissues compared with the adjacent noncancerous tissues; however, the mechanism of upregulation in HCC has not yet been elucidated. In the current study, more attention has been focused on ncRNA regulation. miRNAs were predicted by bioinformatics analysis and transfected into cells to detect their effect on MRP4 expression. Circular RNAs, which act as miRNA sponges, were assessed in HCC cell lines to elucidate the circRNA-miRNA-MRP4 regulatory network.

## 2. Materials and Methods

### 2.1. Patient Samples

A total of 19 paired hepatocellular carcinoma and adjacent noncancerous tissues were obtained from The First Affiliated Hospital Zhejiang University (Hangzhou, China). Patients’ clinical information is listed in Supplementary Materials Table S1. The whole study was approved by the ethics committee of the college of Pharmaceutical Sciences, Zhejiang University (No. 004).

### 2.2. Cell Lines

Human primary hepatocyte cells and several HCC cell lines (SMMC-7721, Huh7, HepG2 and Hep3B) were chosen for study. Human primary hepatocyte cells were obtained from the Research Institute for Liver Diseases (Shanghai) Co. Ltd. (Shanghai, China). SMMC-7721 was kindly gifted by the Department of Pharmacology, College of Pharmaceutical Science, Zhejiang University. Huh7 and HepG2 were obtained from the China Center for Type Culture Collection (Wuhan, China). Hep3B was kindly provided by Stem Cell Bank, Chinese Academy of Sciences (Shanghai, China). Dulbecco’s modified Eagle’s medium (GIBCO, Grand Island, NY, USA) was used for SMMC-7721, Huh7 and HepG2 and Eagle’s Minimum Essential medium (GIBCO) was used for Hep3B. Supplemented with 10% fetal bovine serum (FBS, GIBCO), cells were maintained in a humidified atmosphere with 5% CO_2_ at 37 °C.

### 2.3. Cell Transfection

siRNA for circHIPK3 (si-circHIPK3) and miR-124-3p mimics, miR-4524-5p mimics, miR-124-3p inhibitors, miR-4524-5p inhibitors and their corresponding controls were purchased from Shanghai GenePharma company (Shanghai, China). The sequences used are listed in Supplementary Materials Table S2. Transfection reagent (Polyplus, jetPRIME) was applied. The transfection concentration per well in 24-well plates was 100 nM for si-circHIPK3, miR-124-3p mimics, miR-4524-5p mimics, miR-124-3p and miR-4524-5p inhibitors and their corresponding controls. Transfected cells were collected for qRT-PCR analysis, Western blot and other cell behavior experiments.

### 2.4. qRT-PCR Analysis

Total RNA was extracted from cells and tissues using an RNA extraction kit (Axygen, Union City, CA, USA). For the quantification of MRP4 and circHIPK3 mRNA, 500 ng RNA was reverse transcribed into cDNA using a Prime Script RT reagent Kit (DRR036A, Takara, Shiga, Japan). GAPDH and UBC were separately used as an endogenous reference for cells and tissues. For miR-124-3p and miR-4524-5p quantification, miR-124-3p- or miR-4524-5p-specific stem-loop primers were applied to perform reverse transcription (DRR037A, Takara, Japan) and U6 was used as an endogenous reference. qPCR was performed on an ABI Stepone plus Fast Real-Time PCR system (Applied Biosystems, Waltham, MA, USA) using SYBR Premix Ex Taq II (Takara, Shiga, Japan) and the relative expression of targets was calculated by a 2^−ΔΔCt^ method. Specific primers were listed in Supplementary Materials Table S3.

### 2.5. Western Blot

Total protein was extracted from cells and tissues using radioimmunoprecipitation assay (RIPA) lysis buffer. Proteins were separated on 10% sodium dodecyl sulphatepolyacrylamide gels and transferred to polyvinylidene difluoride membranes. After blocking for 1 h with 5% milk powder at room temperature, the membranes were incubated with anti-MRP4 (1:500) (Abcam, Cambridge, UK) and anti-GAPDH (1:5000) (Proteintech dilution) at 4 °C overnight, respectively. Then, the membranes were incubated with horseradish peroxidase (HRP)-conjugated antirat or antimouse IgG secondary antibodies (1:5000) for 2 h (MultiSciences (LianKe) Biotech Co., Hangzhou, China). An enhanced chemiluminescence (ECL) detection kit (Beijing 4A, Beijing, China) was applied and gray intensity analysis was performed using Image J software (1.46r, NIH, Bethesda, MD, USA).

### 2.6. Luciferase Reporter Assay

A luciferase reporter assay was applied to validate the binding of miR-124-3p and miR-4524-5p to MRP4 3‘-UTR or circHIPK3. The fragments of MRP4 3′-UTR and seed regions of miR-124-3p mutants were cloned downstream of psiCHECK-2 plasmids with firefly luciferase as the standard. To characterize the actual target site of miR-124-3p or miR-4524-5p in circHIPK3, we designed the mutated versions of the original binding site sequences. A single mutation in each of the four miR-124-3p and the two miR-4524-5p binding sites was introduced and inserted into psiCHECK-2 plasmids with firefly luciferase as a control reporter for normalization. All relevant primers are listed in Supplementary Materials Table S4. HEK293T cells were cotransfected with wild type or mutated vectors and miR-124-3p or miR-4524-5p mimics, respectively, for 24 h. A dual-luciferase Reporter Assay (Promega (Beijing) Biotech Co., E1910, Beijing, China) was performed according to the manufacturer’s instructions.

### 2.7. circRNAs In Vivo Precipitation (circRIP)

CircRIP assay was performed as described [23]. Separately, SMMC-7721 and Huh7 were plated into 10 cm dishes and transfected with 1 μL circhipk3 biotin-labeled probes (1 mM) and oligo probes as a negative control for 48 h. The probe sequences are listed in Supplementary Materials Table S5. Transfected cells were fixed with 1% formaldehyde for 10 min, and glycine solution was added to terminate the fixation for 5 min. Then, cells were scratched and lysed for 10 min. Next, the lysates were sonicated and centrifugated at 10,000× *g* for 10 min. A total of 50 μL of the supernatant was preserved as the input. Later, circHIPK3-specific biotin labeled probes–streptavidin beads mixture was pretreated and incubated with the remaining part overnight at 4 °C. On the next day, after reversing the formaldehyde crosslinking, total RNA was extracted by adding Trizol (Takara) and was condensed with Dr.GenTLE^TM^ Precipitation Carrier (Takara). The contents of circHIPK3, miR-124-3p and miR-4524-5p mRNA were measured by RT-qPCR assay.

### 2.8. Statistical Analysis

All data are shown as mean ± standard error of the mean (SEM) except when otherwise stated. Wilcoxon test was applied to detect the differences between tumor tissues and paired adjacent tissues using spss25 (IBM, SPSS, Chicago, IL, USA). Differences between the two experimental groups were analyzed by the Student’s t test. The differences were calculated and *p* < 0.05 was regarded as significant. Survival analysis was performed by Kaplan–Meier curves and log-rank test for significance in GraphPad Prism 5.0 (GraphPad software, Inc., San Diego, CA, USA).

## 3. Results

### 3.1. MRP4 Is Upregulated in HCC

**Figure 1:**
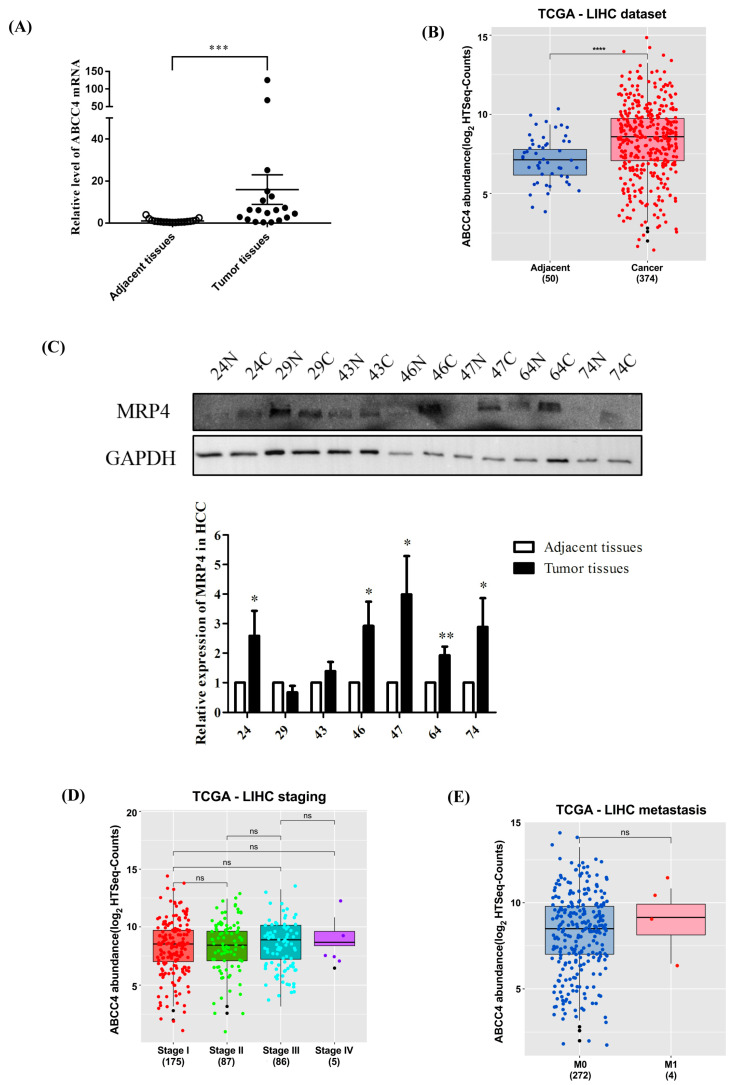
Multidrug resistance-associated protein 4 (MRP4) is upregulated in hepatocellular carcinoma (HCC). (**A**) The RT-qPCR results showed significant upregulation of *ABCC4* mRNA in HCC tissues compared to the adjacent noncancerous tissues. Ubiquitin C (UBC) was used as reference, *n* = 19. Wilcoxon test was applied to detect the differences between tumor tissues and paired adjacent tissues. * *p* < 0.05 (**B**) *ABCC4* expression levels in TCGA database (*n* = 50 for adjacent group; *n* = 374 for cancer group). (**C**) Western blotting analyses of seven randomly selected paired tissues and the expression of MRP4 was normalized to glyceraldehyde-3-phosphate dehydrogenase (GAPDH). N, normal; C, cancer. Results are expressed as mean ± standard error of the mean (SEM) of at least three independent experiments. * *p* < 0.05, ** *p* < 0.01. (**D**) *ABCC4* expression levels of different stages in TCGA database (*n* = 175 for Stage I; *n* = 87 for Stage II; *n* = 86 for Stage III; *n* = 5 for Stage IV). (**E**) *ABCC4* expression levels of nonmetastatic and metastatic HCC in TCGA database (*n* = 272 for M0; *n* = 4 for M1). Differences between two experimental groups were analyzed by the Student’s *t* test., * *p* < 0.05, ** *p* < 0.01, *** *p* < 0.001, **** *p* < 0.0001. “ns” stands for not significant.

MRP4 with 1325 amino acids is the shortest member of the adenosine triphosphate binding cassette subfamily C, which is encoded by the *ABCC4* gene. The RT-qPCR results showed significant upregulation of *MRP4/ABCC4* mRNA in HCC tissues compared to the adjacent noncancerous tissues (Figure 1A), which was in agreement with the expression patterns retrieved from the The Cancer Genome Atlas Program (TCGA) (Figure 1B). Further, Western blotting analysis of seven randomly selected paired tissues (Figure 1C) indicated higher expression of MRP4 protein in HCC tissues, which was consistent with its mRNA expression. The expression of MRP4 protein was found to be comparatively lower in a few HCC tissues, which may be attributed to the individual genetic variations of the patients. As shown in Figure 1D, there was no significant difference in the four stages of HCC. It was also declared no difference in nonmetastatic and metastatic HCC in the TCGA database (Figure 1E). Therefore, it can be concluded that there was no effect of MRP4 expression on HCC development and metastasis.

### 3.2. miR-124-3p Directly Regulates the Expression of MRP4 in HCC

**Figure 2:**
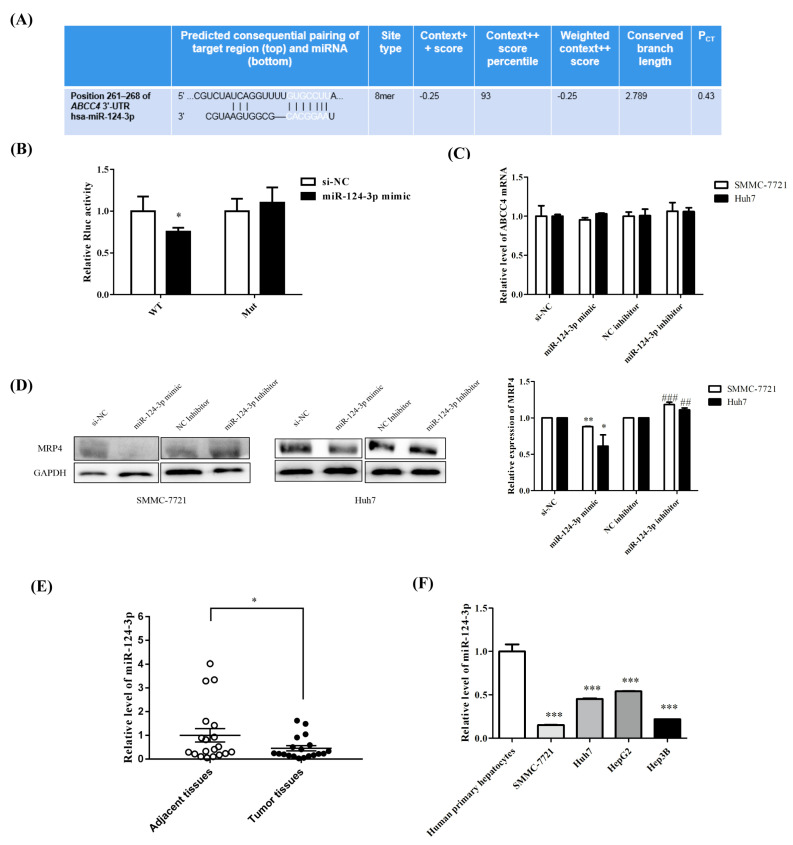
miR-124-3p regulated the expression of MRP4. (**A**) Interaction diagrams between miR-124-3p and *ABCC4* 3′-UTR by computational prediction. (**B**) Luciferase reporter results showed that miR-124-3p could bind directly with *ABCC4* 3′-UTR.si-NC stands for negative control. (**C**) mRNA levels of *ABCC4* was similar in miR-124-3p mimic transfected cells and control cell lines. (**D**) MRP4 protein expression was reduced in SMMC-7721 and Huh7 cells transfected with 100 nM miR-124-3p mimics for 72 h, while treatment with the miR-124-3p inhibitors had contrasting effects. Results are expressed as mean ± standard error of the mean (SEM) of at least three independent experiments. * *p* < 0.05, ** *p* < 0.01, *** *p* < 0.001, compared with control group. ## *p* < 0.01, ### *p* < 0.001, compared with NC inhibitor group. (**E**) RT-qPCR results showed downregulation of miR-124-3p in HCC tissues compared to the adjacent noncancerous tissues. U6 was used to normalize the miRNA expression, *n* = 19. (**F**) Significant downregulation of miR-124-3p was observed in HCC cell lines compared to that in the human primary hepatocytes. Differences between two experimental groups were analyzed by the Student’s *t* test. * *p* <0.05, ** *p* <0.01, *** *p* <0.001.

MiRNAs that can bind to the 3′-UTR of *ABCC4* were predicted by the Targetscan database (http://www.targetscan.org/vert_72/, accessed on 18 January 2021) (Figure 2A). The wild-type (WT) and mutant (Mut) 3′-UTR sequences of *ABCC4* containing a putative binding motif of miR-124-3p were designed. To further validate the binding of these miRNAs to MRP4, wild-type (WT) and mutant (Mut) 3′-UTR sequences of *ABCC4* were cloned into separate psiCHECK-2 plasmids. A luciferase reporter assay was performed to validate the binding of miR-124-3p to MRP4. Compared to the negative control (NC), the renilla luciferase activity in the wild-type group was significantly lower upon miR-124-3p overexpression. Further, introducing mutations in the putative miR-124 binding site abolished the alteration in renilla luciferase activity (Figure 2B). *ABCC4* mRNA expression was also found to be higher in HCC cell lines than that in human primary hepatocytes (Supplementary Materials Figure S1). Among the cell lines tested, SMMC-7721 and Huh7 were selected for further investigation. Similar mRNA levels of *ABCC4* were found in miR-124-3p mimics transfected cells and control cell lines (Figure 2C). Furthermore, as demonstrated by Western blot assay, MRP4 protein expression was shown to be reduced in SMMC-7721 and Huh7 cells transfected with 100 nM miR-124-3p mimics for 72 h, while treatment with the miR-124-3p inhibitors had contrasting effects (Figure 2D). Further, the RT-qPCR results showed downregulation of miR-124-3p in HCC tissues compared to the adjacent noncancerous tissues (Figure 2E). However, significant downregulation of miR-124-3p was observed in HCC cell lines compared to that in the human primary hepatocytes (Figure 2F). HCC cells were cloned from human HCC tissues, while primary hepatocytes were isolated from normal human liver tissues. Hence, the abundance of miR-124-3p in these two types of cells may provide clues to help identify the expression level of miR-124-3p in human liver tissues. Together, these results indicate that miR-124-3p decreased the expression of MRP4 by binding to the 3′-UTR sequence of *ABCC4*.

### 3.3. circHIPK3 Interacted with miR-124-3p and a New Binder miR-4524-5p

**Figure 3:**
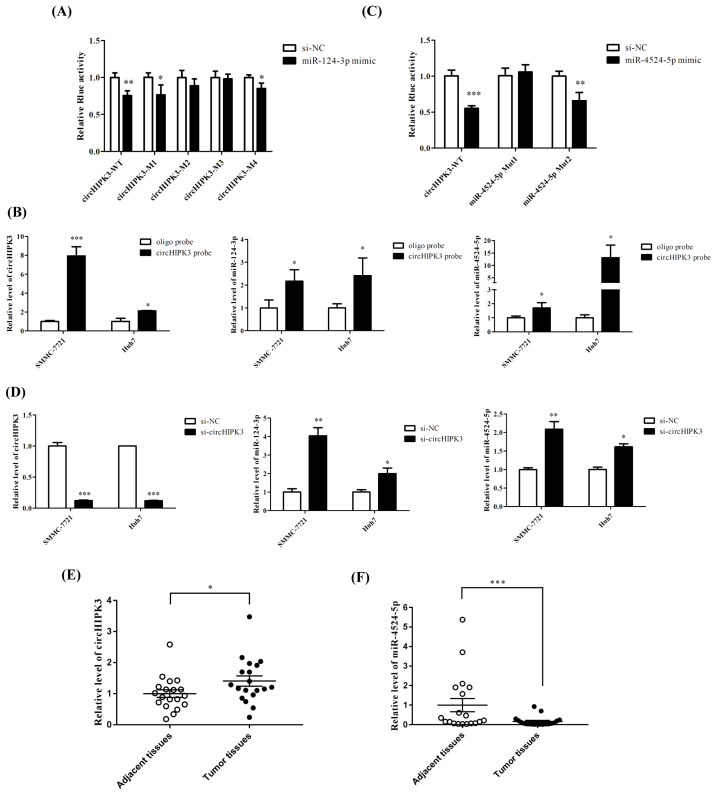
circHIPK3 interacted with miR-124-3p and miR-4524-5p. (**A**) Luciferase assay identified interaction between circHIPK3 and miR-124-3p. (**B**) circHIPK3 was highly enriched and both miR-124-3p and miR-4524-5p were detected in the precipitate in the probe group. Oligo probe was negtive control. (**C**) Luciferase assay identified interaction between circHIPK3 and miR-4524-3p. (**D**) Knockdown of circHIPK3 resulted in the corresponding increase in miR-124-3p and miR-4524-5p. (**E**) The expression of miR-4524-5p was found to be downregulated in HCC tissues than paired adjacent normal tissues. (**F**) The expression of circHIPK3 was found to be significantly higher in HCC tissues than paired adjacent normal tissues, *n* = 19. Differences between two experimental groups were analyzed by the Student’s t test. Wilcoxon test was applied to detect the differences between tumor tissues and paired adjacent tissues. * *p* < 0.05, ** *p* < 0.01, *** *p* < 0.001, compared with control group.

circHIPK3 is an oncogene and is known to be involved in various cellular physiological and pathological processes. Recent studies have shown that circHIPK3 functions as a competitive endogenous RNA (ceRNA) and sponges multiple miRNAs. Through prediction, we identified the binding sites of miR-124-3p in circHIPK3 (hsa_circ_0000284), as shown in Supplementary Materials Figure S2. As shown in Figure 3A, renilla luciferase activity was reduced in HEK293T cells cotransfected with psiCHECK-2-circHIPK3-WT and miR-124-3p mimics compared to the cells that were cotransfected with psiCHECK-2-circHIPK3-MUT and siRNA-NC mimics. Further, the renilla luciferase activity was restored upon transfection of the binding site sequences mutated at sites 2 and 3 rather than sites 1 and 4. To further confirm the interaction between circHIPK3 and miRNAs, we purified the circHIPK3-associated RNAs by circRIP and analyzed the expression of miRNAs. Compared to the oligo probe group (a negative control), higher enrichment of circHIPK3 was found in the circHIPK3 probe group. Further, miR-124-3p was detected in the precipitate of the probe group and miR-4524-5p was also detected (Figure 3B), indicating the binding between the miRNAs and circHIPK3. In the same way, miR-4524-5p binding sites in circHIPK3 were also predicted (Supplementary Materials Figure S3) and the luciferase reporter assay preliminarily verified their interaction. After transfection of the HEK293T cells with miR-4524-5p mimics along with the corresponding WT and Mut binding site sequences, the renilla luciferase activity was found to be reduced in cells transfected with psiCHECK-2-circHIPK3-WT and psiCHECK-2-circHIPK3-MUT2, but not with psiCHECK-2--circHIPK3-MUT1, suggesting that miR-4524-5p binds to circHIPK3 only at site 1 (Figure 3C). Moreover, expression of circHIPK3 was found to be significantly decreased by siRNA knockdown, resulting in corresponding increases in miR-124-3p and miR-4524-5p levels (Figure 3D). These data suggest that circHIPK3 directly interacts with miR-124-3p and miR-4524-5p in SMMC-7721 and Huh7 cells. Further, the expression of circHIPK3 was found to be significantly higher in HCC tissues than paired adjacent normal tissues, as indicated by RT-qPCR results (Figure 3E), while miR-124-3p and miR-4524-5p decreased in HCC tissues compared to the adjacent noncancerous tissues (Figure 2E and Figure 3F). MiR-4524-5p has been little investigated. It was firstly validated that miR-4524-5p can bind with circHIPK3 and its expression is affected by circHIPK3.

### 3.4. circHIPK3-miR-124-3p/miR-4524-5p Axis Is Involved in MRP4 Upregulation

**Figure 4:**
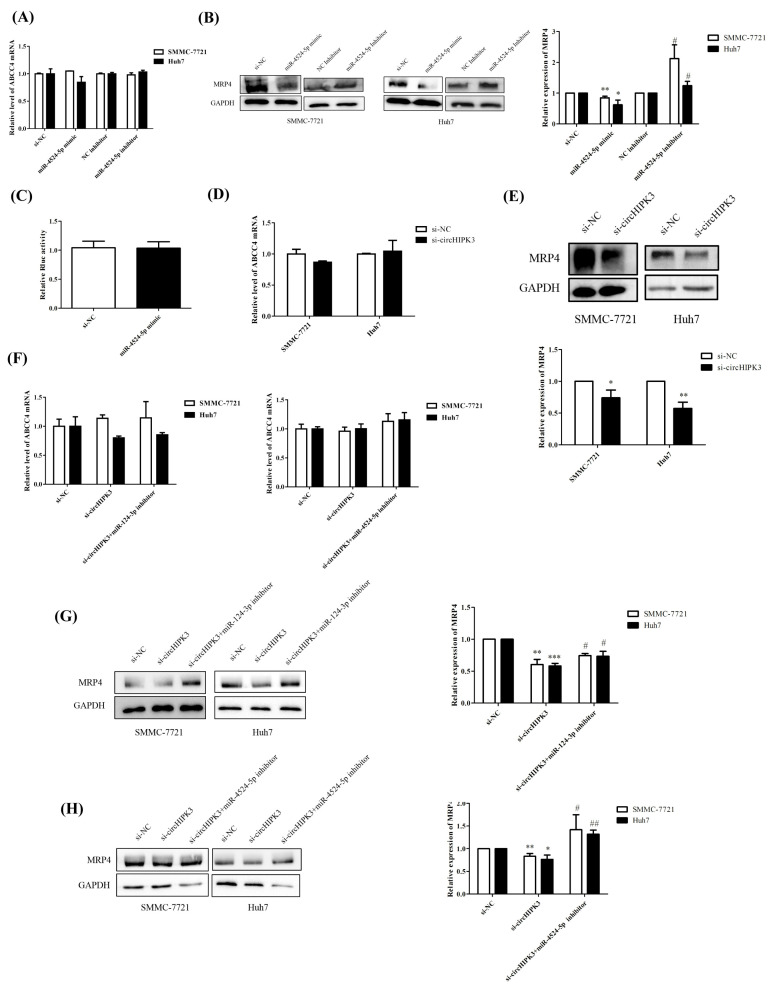
circHIPK3-miR-124-3p/miR-4524-5p axis involved in MRP4 upregulation. (**A**,**B**) Overexpression of miR-4524-5p decreased the MRP4 expression at the protein but not mRNA level. (**C**) No significant difference between the luciferase activity of miR4524-5p and negtive control. (**D**,**E**) Silencing of circHIPK3 decreased the MRP4 expression at the protein but not mRNA level. (**F**) Cotransfection with si-circHIPK3 and miR-124-3p or miR4524-5p inhibitors showed no change in MRP4 mRNA levels. (**G**,**H**) The circHIPK3-associated suppression of MRP4 at the protein level was rescued by inhibition of miR-124-3p or miR-4524-5p.“si-circHIPK3+miR-124-3p inhibitor” stands for cotransfection with si-circHIPK3 and miR-124-3p. “si-circHIPK3+miR-4524-5p inhibitor” stands for cotransfection with si-circHIPK3 and miR-4524-5p. Results are expressed as mean ± standard error of the mean (SEM) of at least three independent experiments. Differences between two experimental groups were analyzed by the Student’s t test. * *p* < 0.05, ** *p* < 0.01, *** *p* < 0.001, compared with control group. # *p* < 0.05, ## *p* < 0.01.

miR-4524-5p mimics were also transfected into SMMC-7721 and Huh7 cells to explore the impact on MRP4. There is no change in the MRP4 mRNA levels after transfection (Figure 4A), which is similar to miR-124-3p. Results of Western blot assay have shown that MRP4 protein expression decreased after transfection with miR-4524-5p mimics, while treatment with its inhibitors had the reverse effects (Figure 4B). These results show that miR-4524-5p regulated MRP4 in the levels of protein, not mRNA. Unfortunately, no binding site for miR-4524-5p in the 3′-UTR of MRP4 was predicted. The luciferase reporter assay has also demonstrated that miR-4524-5p mimics did not result in the reduction in renilla luciferase activity (Figure 4C). miR-4524-5p did not bind directly to MRP4, but decreased its expression, which indicates that miR-4524-5p may regulate MRP4 expression through post-translational mechanisms.

The above results indicated that miR-124-3p and miR-4524-5p suppress MRP4 expression and are directly targeted by circHIPK3. On the basis of this, we propose that circHIPK3 serves as a miRNA sponge and triggers the downregulation of MRP4 in HCC. Furthermore, silencing of circHIPK3 decreased the MRP4 expression at the protein but not mRNA level (Figure 4D,E). We performed rescue experiments to investigate if circHIPK3 regulates MRP4 expression by sponging miR-124-3p or miR-4524-5p. Cotransfection of HEK293T cells with circHIPK3-specific siRNA (si-circHIPK3) and miR-124-3p or miR4524-5p inhibitors showed no change in MRP4 mRNA levels (Figure 4F). However, the circHIPK3-associated suppression of MRP4 at the protein level was rescued by inhibition of miR-124-3p or miR-4524-5p (Figure 4G,H). Collectively, these findings indicate that circHIPK3 regulates MRP4 expression via sponging miR-124-3p or miR-4524-5p.

## 4. Discussion

Expression of MRP4 in the prostate, lung, muscle, pancreas, testis, ovary, bladder, gallbladder has been reported to be relatively higher than liver [24]. Further, MRP4 is found to be overexpressed in several cancers, such as pancreatic cancer [25], prostate cancer [26], acute myeloid leukemia [27], as well as nonsmall cell lung cancer [28]. In the current study, MRP4 was found to be upregulated in HCC compared to the noncancerous tissues both at the mRNA and protein levels. Due to the heterogeneity of tissue specimens, it is possible that the expression of ABCC4 in tumor tissues is different, which was higher than that in paired adjacent tissues. MRP4 is localized in the basolateral membranes of hepatocytes and facilitates the transport of substance from hepatocytes to blood [29]. Therefore, this study focused on the upregulation mechanism of MRP4 expression in HCC.

miRNAs are known to be conserved across species. These small RNAs regulate expression of the targeted genes via binding to their mRNA at 3′-UTR and are reported to be involved in various cellular processes. The regulation of the target genes occurs either by inhibition of translation or degradation of the target mRNA depending on incomplete or complete base pairing of the miRNA with the 3′-UTR of target mRNA. In the present study, we predicted the binding sites of miR-124-3p in the 3′-UTR of *ABCC4* gene using the TargetScan database. The results from the luciferase reporter assay revealed that miR-124-3p directly binds to the 3′-UTR of *ABCC4*, which is in agreement with results from computational predictions. miR-124-3p significantly reduced MRP4 protein expression without affecting its mRNA levels in SMMC-7721 and Huh7 cells, suggesting that miRNA inhibited MRP4 translation instead of degrading its mRNA. These results are in agreement with the findings reported previously by Markova [30].

CircRNAs play an important role in the development of cancer and are reported to be differentially expressed in various human tumors. The functions of circRNAs are diversified: acting as sponges for miRNA and RNA binding proteins, regulating gene transcription and expression, coding for proteins and other yet unexplored functions. circHIPK3 has been reported as an oncogene as well as a tumor suppressor gene, which promotes and inhibits the proliferation, invasion, and migration of cancer cells, respectively [31,32]. In the current study, knockdown of circHIPK3 increased miR-124-3p activity, indicating the interaction between circHIPK3 and the miRNAs, which was also confirmed by circRIP and luciferase reporter assays. Meanwhile, a new binder with circHIPK3 miR-4524-5p was found in the circRIP experiment. A transfection in vitro assay has shown that miR-4524-5p reduced MRP4 protein, not mRNA.

However, it has demonstrated that miR-4524-5p does not bind with MRP4. This indicates that miR-4524-5p may regulate MRP4 indirectly via affecting the expression of other proteins regulating MRP4. ABC transporters are regulated by some signaling pathways. The signaling pathways regulated the activity and the high expression levels of ABC transporters in malignant cells. Multidrug resistance-associated protein 1 expression is under the control of the phosphoinositide 3 kinase/Akt signal transduction network [33]. It is possible that miR-4524-5p decreased MRP4 through signaling pathways. miR-4524-5p was reduced in colon cancer stem cells compared to the nonstem cells. The bioinformatics analysis showed that miR-4524-5p is a key node in the miRNA-pathway-network, which indicates that it may play important roles in cellular processes [34]. MG132 is a proteasome inhibitor. After MG132 treatment, the mass spectrum analysis showed that MRP4 had ubiquitin binding sites, which suggested that MRP4 might be degraded by ubiquitination [35]. miR-495 downregulated ABCG2 and ERCC1 via regulation of Ubiquitin-conjugating enzyme E2 C (UBE2C) [36]. miR-4524-5p may down regulate the expression of MRP4 by affecting ubiquitination degradation-related proteins. However, further studies are needed to explore the possible regulatory mechanisms associated with these miRNAs.

Furthermore, ablation of circHIPK3 reduced MRP4 expression, which was reversed by transfection of cells with miRNA inhibitors. These data revealed that circHIPK3-miR-124-3p and circHIPK3-miR-4524-5p axes are involved in the regulation of MRP4 expression in HCC. circHIPK3 has been reported to be associated with cisplatin resistance in bladder cancer [37] and gemcitabine resistance in pancreatic cancer [38]. The association between circHIPK3 and multidrug resistance in HCC could also be explored.

**Figure 5:**
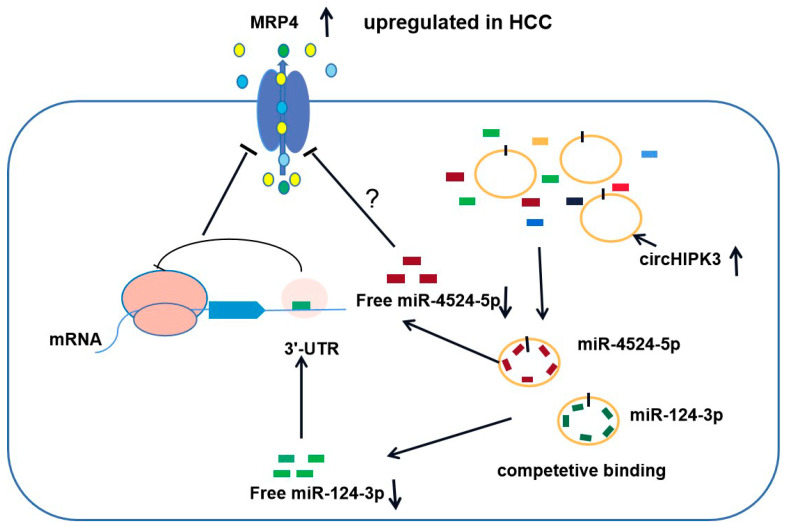
The proposed molecular mechanism of how circRNA regulates the expression of MRP4. MRP4 is upregulated in HCC. miR-124-3p and miR-4524-5p reduced the expression of MRP4 at the protein but not mRNA level. circHIPK3, as a competitive endogenous RNA, binds with miR-124-3p and miR-4524-5p to decrease free miRNAs, which leads to MRP4 upregulation in HCC.

In this study, for the first time, miR-4524-5p was identified to suppress the efflux transporter MRP4 and circHIPK3 regulates the expression of MRP4 through sponging miR-124-3p and miR-4524-5p (Figure 5). Understanding the circHIPK3-miR-124-3p/miR-4524-5p-MRP4 axis may provide new insights into proliferation and MDR in HCC and aid in future studies.

## Data Availability

The data presented in this study are available on request from the corresponding authors.

## Supplementary Materials

The following are available online at https://www.mdpi.com/article/10.3390/biomedicines9050497/s1, Figure S1: MRP4 mRNA expression in human primary hepatocytes and HCC cell lines, Figure S2: Schematic diagram demonstrating the complementary bond within miR-124-3p and circHIPK3 with binding sites predicted by bioinformatics programs, Figure S3: Schematic diagram demonstrating the complementary bond within miR-4524-5p and circHIPK3 with binding sites predicted by bioinformatics programs, Table S1: Patients’ clinical information, Table S2: The siRNA and miRNA sequences, Table S3: The primer sequences for real-time PCR, Table S4: The primer sequences for luciferase reporter plasmids construction, Table S5: The probe sequences for circRIP assay.
