# Supplementary material for: Regulation of MRP4 Expression by circHIPK3 via Sponging miR-124-3p/miR-4524-5p in Hepatocellular Carcinoma
Source: Biomedicines. 2021 Apr 30;9(5):497. doi: 10.3390/biomedicines9050497 (PMC8147194; doi:10.3390/biomedicines9050497)
Supplement: Supplementary file 1 [file biomedicines-09-00497-s001.zip › biomedicines-1192843-supplementary.pdf]

biomedicines

Supplementary Materials

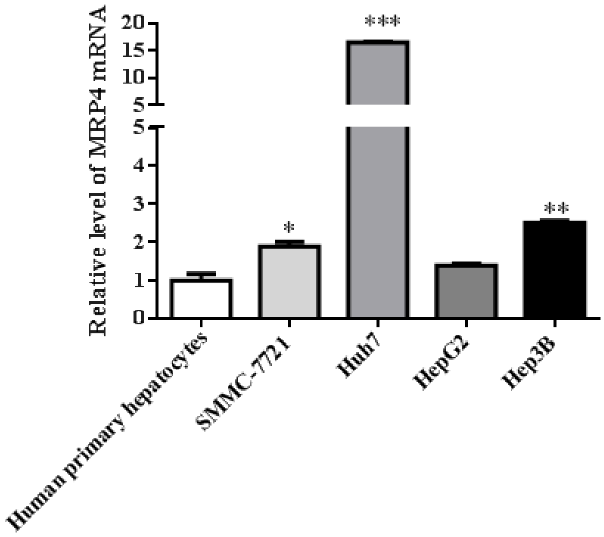

Supplementary Materials Figure S1. MRP4 mRNA expression in human primary hepatocytes and HCC cell lines. * $p < 0.05$, ** $p < 0.01$, *** $p < 0.001$.

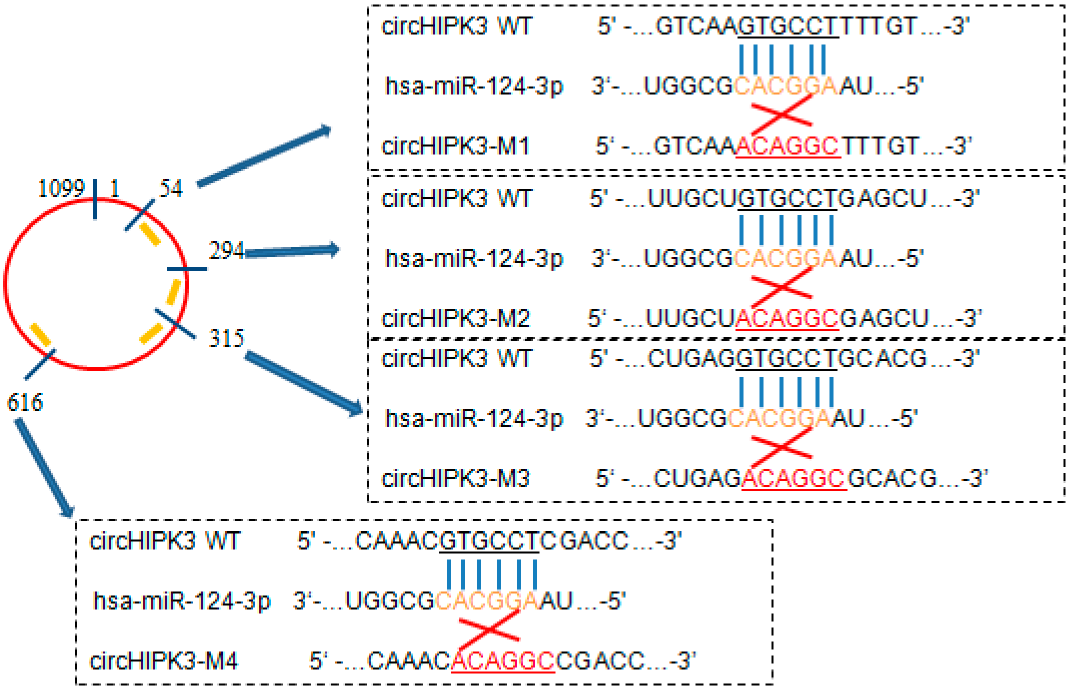

Supplementary Materials Figure S2. Schematic diagram of the complementary bond within miR-124-3p and circHIPK3 with binding sites predicted by bioinformatics programs.

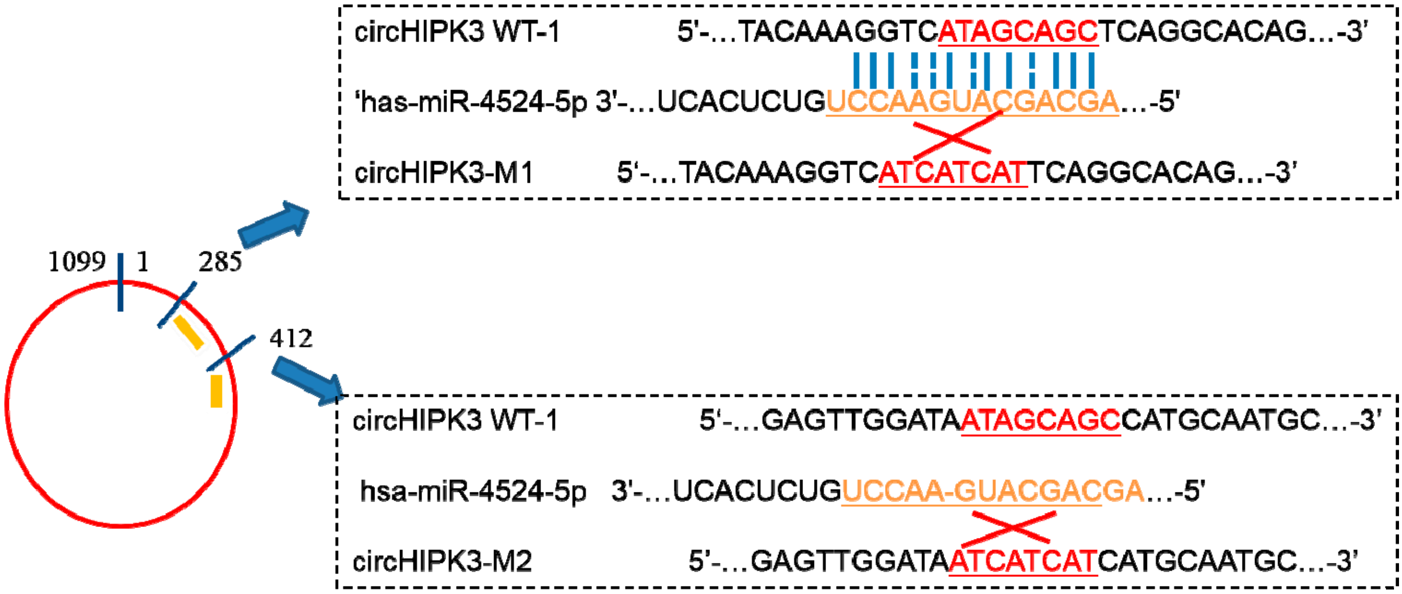

Supplementary Materials Figure S3. Schematic diagram of the complementary bond within miR-4524-5p and circHIPK3 with binding sites predicted by bioinformatics programs.

Supplementary Materials Table S1. Patients' clinical information.

Variable	Total	MRP4 Upregulation	miRNA Downregulation		CircHIPK3 Upregulation
			miR-124-3p	miR-4524-5p	
Age					
≤60	9	8	7	8	8
>60	9	8	5	8	7
Gender					
Male	13	12	9	11	11
Female	6	5	4	6	4
Type					
HCC	14	12	10	13	11
ICC	3	3	2	2	3
Others	2	2	1	2	1
Stage					
I–II	8	7	6	7	5
III–IV	11	10	7	10	10

Supplementary Materials Table S2. The siRNA and miRNA sequences.

**siRNA**	
si-ABCC4#1	ACAGAAGCCUUCUUUAACAdTdT
si-ABCC4#2	UUGCCUAUGUGCUUCAAGAdTdT
si-circHIPK3	CUACAGGUAUGGCCUCACAdTdT
**miRNA**	
miR-124-3p mimics	UAAGGCACGCGGUGAAUGCCAA
miR-4524-5p mimics	AGCAGCAUGAACCUGUCUCACU

Supplementary Materials Table S3. The primer sequences for real-time PCR.

	Sense	Antisense
ABCC 4 mRNA	GGATCCAAGAACTGATGAG-TTAAT	TCACAGTGCTGTCTCGAAAATAG
circHIPK3 mRNA	TATGTTGGTGGATCCTGTTCGG-CA	TGGTGGGTAGACCAAGACTT-GTGA
GAPDH mRNA	AGGTGAAGGTCGGAGTCA	GGTCATTGATGGCAACAA
UBC mRNA	CCTGGTGCTCCGTCTTAGAG	TTTCCCAGCAAAGA TCAACC
miR-124-3p stem-loop primer	GTCGTATCCAGTGCAGGGTCCGAGGTATTCGCACTGGATAC-GACTTGGCA	
miR-124-3p qPCR	CGCCTAAGGCACGCGGTGAA	GTGCAGGGTCCGAGGT
miR-4524-5p stem-loop primer	TCATGCTGCTCTATGCTCTCCAGGTACAGTTGGTACCTGTCTCCAC-TTAGTGAG	
miR-4524-5p qPCR	TCATGCTGCTCTATGCTCTCCAG	GTGCAGGGTCCGAGGT
U6 stem-loop primer	GTCGTATCCATGGCAGGGTCCGAGGTATTCGCCATGGATACG	
U6 qPCR	CTCGCTTCGGCAGCACA	AACGCTTCACGAATTTGCGT

Supplementary Materials Table S4. The primer sequences for luciferase reporter plasmids construction.

	Sense	Antisense
ABCC4 3'-UTR	CCGCTCGAGATCCAAC-CAAAATGTCAAGTCC	TAAAGCGGCCGCCTGAATGGA-GATGAAAACTATCAT
ABCC4-miR-124-3p Mut	GCCGTC-TATCAGGTTTTACAGGCTAA-GAGACTACAGAGTCAAAGC	GCTTTGACTCTGTAG-TCTCTTAGCCTGTAAAACCTGA-TAGACGGC
circHIPK3-WT	TATCTCGAGGTATGGCCTCACA AGTC	TAAAGCGGCCGCCTGTAGTAC-CGAGATTGTAG
circHIPK3-miR-124-3p-M1	CCATATGTTTATCAAACTCAG-TCAAACAGGCTTTGTAG-TGTGAAGAAACTCAAAG	CTTTGAGTTTCTTCACAC-TACAAAGCCTGTTTGACTGAG-TTTGATAAACATATGG
circHIPK3-miR-124-3p-M2	CAAAGGTCATAGCAGCTCGCCT GTAGCAAGCTCACGTG	CACGTGAGCTTGCT ACAGGC GAGCTGCTATGACCTTTG
circHIPK3-miR-124-3p-M3	GCAAGCTCAC-GTGCGCCTGTCTCAGATTGGGG CGT	ACGCCCCAATCTGAG ACAGGC GCACGTGAGCTTGC
circHIPK3-miR-124-3p-M4	CCTTGATTTTCTT-GGTCGGCCTGTGTTT-GGCCAGGTAGTTAAAT	ATTTAACTACCTGGCCAAAC ACAGGC CGACCAA-GAAAATCAAGG
circHIPK3-miR-4524-5p-M1	CAGGTGC-TAC-AAAGGTCATCATCATTCAGGCA CAGCAAGC	GCTT-GCTGTGCCTGAATGATGATGAC-CTTTGTAGCACCTG
circHIPK3-miR-4524-5p -M2	GAGTGAGGAGTTGGA-TAATCATCATCATGCAATGCAG ATTGTCGA	TCGACAATCTGCATT-GCATGATGATGAT-TATCCAACTCCTCACTC

Supplementary Materials Table S5. The probe sequences for circRIP assay.

	Sequence (5'-3')
circHIPK3 probe	CCAAGACTTGTGAGGCCATACCTGTAGTACCGAGATTGTAGATATGTT-GAA
Oligo probe	CTTCAAGACTGTGCCAAATTTAGAAAATATCAAAAGCAGCAGCCA-TAGGG
